# Abschied vom Recht auf Bildung? Bildungsbenachteiligung von Kindern durch Schulschließungen während der Covid-19-Pandemie

**DOI:** 10.1007/s12054-022-00465-4

**Published:** 2022-03-29

**Authors:** Carolin Butterwegge, Christoph Butterwegge

**Affiliations:** Universität zu Köln, Köln, Deutschland

**Keywords:** Covid-19, Corona-Pandemie, Schulschließungen, Soziale Ungleichheit, Bildungsungleichheit

## Abstract

Schulschließungen während der Covid-19-Pandemie haben nicht bloß zu Lernrückständen bei vielen Kindern und Jugendlichen geführt. Vielmehr wuchs auch die in der Bundesrepublik aufgrund der wachsenden sozialen Ungleichheit die ohnehin stark ausgeprägte Bildungsungleichheit. Hauptleidtragende der monatelangen Kita- und Schulschließungen waren Kinder aus einkommensschwachen Familien.

Um das Recht auf Bildung für sämtliche Kinder und Jugendliche zu gewährleisten, haben sich alle UN-Mitgliedstaaten bis auf die USA in der Kinderrechtskonvention von 1989 verpflichtet, den regelmäßigen Schulbesuch zu fördern. Während der Covid-19-Pandemie konterkarierten aus Gründen des Infektionsschutzes verfügte Kita- und Schulschließungen dieses bildungspolitische Kernziel. Hierunter leiden besonders Minderjährige aus sozial benachteiligten Familien bis heute, weil sie zum Teil regelrecht abgehängt wurden.

Laut einer Untersuchung des Bundesinstituts für Bevölkerungsforschung waren die Schulen hierzulande im ersten Lockdown vom 23. März 2020 bis zum 5. Mai 2020 an insgesamt 44 Tagen weitgehend geschlossen. Anschließend erfolgte zwar eine partielle Öffnung der Schulen, in mehreren Bundesländern blieben die Sekundarstufen aber noch länger zu. Teilweise gab es bis zu den Sommerferien nur Wechselunterricht. Durchschnittlich waren die Schulen bis zu den Sommerferien an 59 Tagen partiell geschlossen. Insgesamt fand zwischen eineinhalb und drei Monaten für die meisten Kinder überhaupt kein Präsenzunterricht statt (vgl. Bujard et al. 2021, S. 8).

Während des zweiten Lockdowns im Winter 2020/21, der als kurzer „Wellenbrecher“ begann, aber mehrfach verlängert und verschärft wurde, blieben die Schulen an insgesamt 61 Tagen fast vollständig zu. Anschließend wurde der Präsenzschulbetrieb insbesondere für Grundschulkinder zum Teil wieder aufgenommen, wobei es im Sekundarschulbereich erhebliche Unterschiede von einem Bundesland zum anderen gab. Bis zum 7. Juni 2021 erfolgten partielle Schulschließungen an 112 Tagen. Insgesamt belief sich deren Dauer bis Anfang Juni 2021 somit auf 173 Tage. Für die Grundschüler_innen betrug die Zeit ohne Präsenzunterricht mindestens zwei Monate. „In einigen Bundesländern waren Schülerinnen und Schüler der siebten bis zwölften Klasse von Mitte Dezember 2020 bis Mitte Mai 2021 nicht in der Schule. Mehrere Millionen Kinder und Jugendliche haben in diesem zweiten Lockdown vier oder fünf Monate keine Schule in Präsenz besucht und diese Monate stattdessen vor dem Computer im Kinderzimmer verbracht und an digitalem Distanzunterricht teilgenommen oder konventionell Aufgaben mit Stift und Papier bearbeitet.“ (ebd.).

Unstrittig ist, dass der wiederholte Lockdown bei einem Großteil der Schüler_innen zu Lernrückständen geführt hat. Außerdem häuften sich Verhaltensauffälligkeiten und psychosoziale Probleme bei den jungen Menschen aufgrund der pandemiebedingten Schulschließungen. Diese führten zusammen mit den Kontaktbeschränkungen und dem Homeoffice vieler Eltern zu einer tiefgreifenden Veränderung des Familienlebens, aber auch der Kindheit selbst.

## Verschärfung der Bildungsungleichheit durch wachsende soziale Ungleichheit

In der Bundesrepublik waren die Bildungschancen ebenso ungleich verteilt wie die Infektionsrisiken. In beiden Fällen spielten die soziale Herkunft und der familiäre Hintergrund von (Schul‑)Kindern eine Hauptrolle (vgl. hierzu: Butterwegge und Butterwegge 2021, S. 169 ff.). Wie im Bildungsbericht 2020 festgestellt wurde, bestand sogar die Gefahr, dass sich die „Schere zwischen sozial benachteiligten und privilegierteren Kindern und Jugendlichen“ aufgrund der Covid-19-Pandemie weiter öffnete; fraglich erscheint jedoch, ob eine weitere Verschärfung der Bildungsungleichheit durch „professionell ausgewählte und begleitete digitale Lehr-Lern-Gelegenheiten“ verhindert werden kann, wie die Autorengruppe Bildungsberichterstattung (2020, S. 150) erwartete.

Dass die Bildungschancen der Kinder maßgeblich vom Sozialstatus, Bildungsniveau und Geldbeutel der Eltern abhängen, ist ein Kardinalproblem des mehrgliedrigen Schulwesens, das sich aufgrund der pandemiebedingten Schulschließungen sowie der während dieser Zeit erprobten Modelle eines Distanz- und Wechselunterrichts (abwechselnder Präsenzunterricht in der Schule und Fernunterricht zuhause) in zweierlei Hinsicht erheblich verschärft hat: Einerseits waren Schulen mit einem hohen Sozialindex, d. h. einem überdurchschnittlich hohen Anteil von Schüler_innen im Hartz-IV-Bezug, nach den vom Land Nordrhein-Westfalen erhobenen COSMO-Daten (Corona-Schnellmeldung online) während des „angepassten Regelbetriebs“ im zweiten Halbjahr 2020 häufiger von (Teil‑)Schließungen betroffen, die einen Übergang vom Präsenz- in den Distanzunterricht erzwangen. Daher befanden sich andererseits überproportional häufig gerade jene Schüler_innen im Distanzunterricht, deren soziale, familiale, wohnliche und technische Voraussetzungen dafür besonders ungünstig waren (vgl. Schräpler et al. 2021, S. 303).

Debatten über mögliche Gründe für die Verschärfung der Bildungsungleichheit durch längere Schulschließungen bergen zwei Gefahren: Erstens wird häufig übersehen, dass es sich dabei um ein Strukturproblem handelt, das auch schon vor der Covid-19-Pandemie bestand; zweitens werden Defizite im Hinblick auf die Ausstattung sozioökonomisch benachteiligter Elternhäuser mit digitaler Technik fälschlicherweise als Hauptursache für die „aufgehende Bildungsschere“ angesehen, wohingegen die qualitativen Unterschiede beim Distanzunterricht unberücksichtigt bleiben, der mancherorts gut und andernorts gar nicht funktioniert hat.

Beobachter_innen überraschte die große, mit einer strukturellen Bildungs- und Teilhabeungerechtigkeit verbundene Heterogenität der schulischen Betreuung (vgl. Christiansen und Steinmayr 2020, S. 60). Da gab es Schulen mit einer überwiegend soziökonomisch benachteiligten Schülerschaft in herausfordernden Lagen, in denen manch eine Lehrkraft wegen vermuteter Ausstattungsmängel der Elternhäuser und/oder von in der Schulkultur verbreiteter Defizitorientierungen im Hinblick auf digitale Kompetenzen der Schüler_innen einen Fernunterricht für ihre mehrheitlich benachteiligten Schüler_innen anfangs gar nicht erst in Betracht gezogen hat. Andere Lehrkräfte stießen schnell an ihre Grenzen, wenn es im Lockdown darum ging, überhaupt Kontakt zu allen Schüler_innen bzw. ihren Elternhäusern herzustellen und aufrechtzuerhalten. Der postalische Versand oder Bring- und Abholdienste von Arbeitsblättern am Wochenbeginn ersetzten weder Rückmeldungen zur Aufgabenbearbeitung noch den sozialen Austausch von Lehrkräften mit Schüler_innen, etwa über das kindliche Wohlbefinden oder dessen Gefährdung. Mehrheitlich bekamen die (älteren) Schüler_innen nach den üblichen Startschwierigkeiten durchaus einen gut organisierten Online-Distanzunterricht angeboten, mit dem sie gut zurechtkamen und sogar Lernfortschritte machten.

Im ersten Lockdown war der Mangel an digitalen Endgeräten eines der Hauptprobleme von Schüler_innen. Auch mussten sich die Schulen mitsamt ihren Lehrkräften erst auf den Distanzunterricht einstellen und ihn organisieren lernen, während digitale und datenschutzkonforme Schulplattformen vielerorts noch nicht etabliert waren. Die üblichen Bildungsangebote trotz geschlossener Schulen aufrechtzuhalten, gelang sehr unterschiedlich und war stark vom Alter der Lerngruppen, ihren Mediennutzungskompetenzen und der sozioökonomischen Lage ihrer Elternhäuser abhängig: Grundschulkinder ohne gefestigte Lese- und Schreibkompetenzen im Distanzunterricht zu motivieren und „mitzunehmen“, erwies sich für Lehrkräfte als am schwierigsten. Daher erhoben Grundschulen neben Abschlussklassen zuerst den Ruf nach Wiederöffnung. Insbesondere an weiterführenden Schulen mit vielen in beengten Wohnverhältnissen lebenden Jugendlichen ohne digitale Endgeräte, Drucker und WLAN sowie mit Eltern, die sich mit der von ihnen erwarteten schulischen Unterstützung ihrer Kinder überfordert sahen, verloren die Lehrkräfte den Kontakt zu nicht wenigen Schüler_innen mit der Folge, dass für diese schlicht und einfach kein Unterricht mehr stattfand.

Die Aufgabe, sämtliche Schüler_innen mit digitalen Endgeräten auszustatten, damit sie am Distanzlernen teilnehmen konnten, wurde nie gänzlich gelöst. Monatelang lehnten die Jobcenter eine Übernahme der Kosten digitaler Endgeräte für Kinder von Hartz-IV-Bezieher_innen, die sich im Homeschooling befanden, etwa mit der Begründung ab, dass es sich dabei nicht um einen laufenden, sondern einen pandemiebedingten Mehrbedarf handle. Nur aufgrund mehrerer Urteile der Sozialgerichte wurden Laptops oder Tablets mit Zubehör wie einem Drucker als nicht vom Regelsatz gedeckter Sonderbedarf anerkannt. Zwar legte der Bund zusammen mit den Ländern am 15. Mai 2020 ein Sofortprogramm in Höhe von 550 Mio. € für den digitalen Unterricht auf. Damit sollten die Bildungseinrichtungen bedürftigen Schüler-innen einen Zuschuss von 150 € für die Anschaffung entsprechender Geräte gewähren und professionelle Online-Lehrangebote erstellen, wodurch sich jedoch nur unwesentliche Verbesserungen erzielen ließen.

Erst ab 1. Januar 2021 wurde alleinstehenden und alleinerziehenden Hartz-IV-Bezieher_innen im Regelbedarf 38,89 € monatlich für die Handynutzung zugestanden, obwohl sie bereits seit mehreren Jahren zum soziokulturellen Existenzminimum gehört. Auch stieg der Regelbedarf trotz dieser Erhöhung nur um 14 € von 432 auf 446 €. Genau einen Monat später wies die Bundesagentur für Arbeit ihre Jobcenter an, den Anspruch auf Übernahme der Kosten für digitale Endgeräte rückwirkend ab Jahresanfang anzuerkennen. Wenn diese für das Homeschooling benötigt, aber nicht von den Schulen bereitgestellt wurden, war ein Zuschuss in Höhe von bis zu 350 € zu bewilligen. Abgesehen davon, dass dieser Geldbetrag kaum ausreichte, um Geräte von guter Qualität anzuschaffen, löste er auch ein weiteres Problem finanzschwacher Familien nicht: Oft fehlt armen Kindern ein eigenes Zimmer und damit ein ruhiger Arbeitsplatz, der ihnen ein konzentriertes Lernen ermöglichen würde. Mehr als ihre materiell bessergestellten Klassenkamerad_innen waren diese Kinder im Homeschooling überfordert. Deshalb schuf das Distanzlernen noch mehr Lerndistanz ausgerechnet bei Kindern, die man in der (Medien‑)Öffentlichkeit ohnehin als bildungsfern abqualifiziert.

Eltern ohne gute Deutschkenntnisse sahen sich aufgrund sprachlicher Barrieren meist nicht in der Lage, ihren Nachwuchs bei den Lern- und Hausaufgaben zu unterstützen. Hingegen konnten akademisch gebildete Eltern aus der Mittelschicht ihren Kindern viel eher behilflich sein, als Lehrkraftersatz fungieren und den Ausfall schulischen Präsenzunterrichts größtenteils kompensieren, sodass die Sprösslinge materiell besser situierter Familien trotz des Distanzunterrichts durchaus Lernfortschritte verzeichnet haben dürften. Schulschließungen und Distanzunterricht steigerten auf diese Weise das Ausmaß der Bildungsungleichheit.

## Distanzunterricht als Verstärker des Trends zur Digitalisierung von Schulen und Kinderzimmern

Zwar verfügten die meisten Schüler_innen im zweiten Lockdown aufgrund der inzwischen erfolgten Beschaffung von Tablets und anderen Endgeräten durch Bund, Länder und Kommunen zumindest über ein digitales (Leih‑)Endgerät, aber auch dieses Problem war keineswegs endgültig gelöst: Gerade Schulen mit einer benachteiligten Schülerschaft standen vor großen Herausforderungen und konnten nicht alle Schüler_innen bedarfsgerecht mit Endgeräten ausstatten, sodass man weiterhin von Schüler_innen hörte, die allenfalls mit einem Handy auf digitale Unterrichtsangebote und Materialien zurückgreifen konnten. Es gibt Hinweise, dass solche digitalen Ausstattungsmängel besonders auf die (in Sammelunterkünften lebenden) Kinder aus Internationalen oder Vorbereitungsklassen sowie auf Kinder zutrafen, an denen eine diesbezügliche Bedarfsabfrage von Schulen zum Beispiel aufgrund von bestehenden Sprachbarrieren oder von misslungener Kommunikation mit dem Elternhaus vorbeilief. Selbst der postalische Versand von Arbeitsblättern wurde offenbar notgedrungen weiter praktiziert. Auch sind manche Schulen immer noch nicht an ein stabiles WLAN angeschlossen. Trotz eines Digitalisierungsschubs war die Form des Hybridunterrichts mit geteilten Klassen selbst im zweiten Jahr der Pandemie in den wenigsten Schulen durchführbar.

Während des mehrfach unterbrochenen, länger andauernden und erst im April 2021 von der „Bundesnotbremse“ abgelösten Lockdowns mussten Kinder und Jugendliche erneut ausbaden, was die politisch Verantwortlichen an Vorsorgemaßnahmen in Kitas und Schulen versäumt hatten. In den höheren Jahrgangsstufen vor allem der Gymnasien gingen die wochenlangen Schulschließungen größtenteils mit einer überstürzten Digitalisierung des Unterrichts (Homeschooling und E‑Learning) einher, wodurch sich die Benachteiligung von Kindern aus finanzschwachen Familien im Bildungsbereich verstärkt hat. Denn nicht immer waren die Ausstattungsmängel in Bezug auf Technik im eigenen Haushalt befriedigend gelöst, was nötig gewesen wäre, um nicht ins Hintertreffen gegenüber materiell bessergestellten Klassenkamerad_innen zu geraten.

In einer digitalen Zweiklassengesellschaft scheitern zwangsläufig jene Kinder, die gar keinen oder nur einen beschränkten Zugang zum schnellen Internet, zu einem Computer und einem Drucker haben. Wenn sie von sämtlichen Online-Angeboten im Bildungs- und Kulturbereich wie auch von den Gruppenchats ihrer Peergroup ausgeschlossen waren, fühlten sich die Betroffenen wie „Kinder zweiter Klasse“, die einfach nicht dazugehören.

Eine (erfolgreiche) Teilhabe am Distanz‑, Hybrid- und Wechselunterricht setzte nicht bloß einen Internetanschluss, die Ausstattung mit geeigneten Endgeräten und die Fähigkeit zu selbstständigem Lernen, sondern auch Kompetenzen im Umgang mit digitalen Medien voraus. Schon vor der Pandemie waren die computer- und informationsbasierten Kompetenzen der Schüler_innen in Deutschland im internationalen Vergleich mittelmäßig sowie die Ausstattung mit mobilen Endgeräten, der Zugang zu WLAN in der Schule und die Verfügbarkeit von Lernmanagementsystemen, aber auch die Teilnahme von Lehrkräften an Fortbildungsveranstaltungen zu diesem Kompetenzbereich sogar unterdurchschnittlich.

Zugleich deutet sich eine Verstärkung der sozialen Ungleichheit durch eine Ungleichverteilung der digitalen Kompetenzen zwischen Schüler_innen verschiedener Schulformen und nach der sozialen Herkunft an. Laut der International Computer and Information Literacy Study (ICILS), einer international vergleichenden Schulleistungsstudie, lagen die mittleren Kompetenzwerte, die Gymnasiast_innen erreichten, deutlich vor jenen der Achtklässler_innen anderer Schulformen der Sekundarstufe I (vgl. Eickelmann et al. 2019, S. 13 ff.). Für das Erreichen nur der ersten und/oder zweiten von fünf Kompetenzstufen erwies sich das an der Zahl der Bücher im heimischen Haushalt gemessene „kulturelle Kapital“ der Familie als besonders einflussreich: 43,1 % der Achtklässler/innen aus Familien mit niedrigem, aber bloß 18,8 % der Achtklässler/innen aus Familien mit hohem kulturellen Kapital verfügten über geringe rudimentäre bzw. basale Kompetenzen (vgl. Senkbeil et al. 2019, S. 314 ff.). Je nach der sozialen Herkunft fiel die Ungleichheit bei den computer- und informationsbezogenen Kompetenzen der Schüler/innen zudem in Deutschland höher aus als in fast allen anderen Staaten. Verstärkt wird die Ungleichheit durch ein anderes Nutzerverhalten: Sozial privilegierte Kinder und Jugendliche nutzen digitale Medien eher instrumentell, etwa zur Informationsbeschaffung und zum Lernen als zur Selbstdarstellung oder sozial interaktiven Unterhaltungs-Orientierung. Auch verfügen sie offenbar über eine längere Erfahrung, größere Expertise und ein breiteres Spektrum von Nutzungsoptionen.

Durch die Covid-19-Pandemie hat die Digitalisierung der Kinderzimmer, der Schulen und des Unterrichts einen kräftigen Schub erhalten, wurde die Bundesrepublik doch bisher von der IT-Lobby als in dieser Beziehung unterentwickelt hingestellt. Manchmal ist eine Medizin aber schlimmer als die Krankheit, deren Heilung sie bewirken soll. Ausgerechnet während der Covid-19-Pandemie erreichte die „Smartphone-Epidemie“ (Manfred Spitzer) ihren Höhepunkt. Gerald Lembke, Studiengangleiter für Digitale Medien an der Dualen Hochschule Baden-Württemberg Mannheim, und der Wirtschaftsjournalist Ingo Leipner stehen digitalen Medien sowie ihrem Einsatz im Unterricht zumindest für Grundschüler_innen reserviert gegenüber. Beide haben in Buchveröffentlichungen analysiert, welche politökonomischen Interessen dahinterstehen, sich aber auch mit den sozialen Folgen beschäftigt (vgl. Leipner 2020; Lembke und Leipner 2020). High-Tech-Konzerne, die kaum Steuern zahlen und damit den Staaten jene Einnahmen vorenthalten, die etwa für mehr Frühförderungsmaßnahmen zugunsten sozial Benachteiligter verausgabt werden könnten, machen Gewinne mit dem Verkauf digitaler Lernprogramme für arme Kinder, während ihre Topmanager und andere reiche Eltern ihren eigenen Sprösslingen selbst die Handynutzung verbieten.

Aufgrund der Schulschließungen im Zuge der Pandemie sowie der durch diese beschleunigten Digitalisierung des Bildungsbereichs droht eine Vergrößerung der herkunftsbedingten Unterschiede in der gesellschaftlichen Teilhabe von Kindern und Jugendlichen. Denn es kam zu einer digitalen Spaltung der jungen Generation. Die bereits zuvor bestehenden Ungleichheiten der Bildungschancen haben sich in der Krisensituation vertieft und verfestigt, weil den Elternhäusern mehr Einfluss zukam und die meisten Schulen schlecht für die Pandemie gerüstet waren. Die bisherigen Antworten von Staat, Wirtschaft und Gesellschaft auf diese Herausforderungen sind wenig zufriedenstellend. Ungelöst bleibt die für den gesellschaftlichen Zusammenhalt entscheidende Frage, wie man sozial ausgegrenzten und abgehängten Kindern gewissermaßen nachholend bessere Chancen auf eine doch noch erfolgreiche Bildungsteilhabe bieten kann.
